# Assessment of the prevalence of Behcet's disease in recurrent aphthous ulceration worldwide: a systematic review

**DOI:** 10.4317/medoral.27023

**Published:** 2025-03-23

**Authors:** Ana Beatriz Fernandes Azevêdo, Ana Paula Veras Sobral, Caroline Augusta Belo Faria, Juliana Syndia Silva Santos Sousa, Weslay Rodrigues da Silva, Márcia Maria Fonseca da Silveira

**Affiliations:** 1 Department of Oral and Maxillofacial Pathology, School of Dentistry, Postgraduate Program in Dentistry, University of Pernambuco (UPE), Recife, PE, Brazil; 2 Hospital Universitário Oswaldo Cruz (HUOC), Integrated Anatomic Pathology Center, Recife, Pernambuco, PE, Brazil

## Abstract

**Background:**

The aim of this study was to evaluate the prevalence of Behcet's syndrome in patients with recurrent aphthous stomatitis (RAS).

**Material and Methods:**

A search was performed in Scopus, Medline/PubMed, The Cochrane Library and Web of Science databases, according to PRISMA. In addition, a search was carried out in the DANS Easy Archive to access gray literature and a manual search in the reference list of included studies was used as an additional resource to refine the search. Cross-sectional studies in patients with recurrent aphthous stomatitis evaluating the diagnosis of Behcet's syndrome were analyzed to identify prevalence. The Joanna Briggs Institute (JBI) Critical Appraisal Checklist was used to assess the methodological quality of the articles.

**Results:**

The study design, sample size, sex, International Study Group (ISG) diagnostic criteria and prevalence of the syndrome were evaluated. After screening and reading the articles in full, 7 met the inclusion criteria. The articles involved a total of 2841 participants with recurrent aphthous ulcerations, with 141 having a diagnosis of Behcet's syndrome. Studies were of good quality as assessed by the JBI Critical Appraisal Checklist for cross-sectional studies.

**Conclusions:**

The prevalence of Behcet's syndrome in patients with recurrent aphthous stomatitis (RAS) is generally low, with similar frequency in both sexes and more common in the range of countries that extends from the Mediterranean Basin to the Far East.

** Key words:**Behcet's syndrome, aphthous stomatitis, prevalence.

## Introduction

Recurrent aphthous stomatitis (RAS) is a common chronic disease of the oral mucosa, characterized by the recurrence of painful shallow ulcers, often covered with a grayish-white or yellow exudate and surrounded by an erythematous margin, affecting approximately 5 to 25% of the population, although the prevalence can vary from 5 to 60%, depending on the studied population, diagnostic criteria or environmental factors (1). Although some proven factors are related to RAS, such as nutrient deficiency, intestinal flora imbalance, trauma, bad lifestyle habits, psychological factors, immunological diseases and genetic susceptibility, its etiology still remains unknown (2). Among these conditions, Behcet's syndrome (BS) stands out, a rare systemic inflammatory disease that manifests itself as a triad of symptoms: recurrent oral and genital ulcers and associated uveitis (3).

Behcet's syndrome is a systemic vasculitis that affects multiple organs, involving the skin, mucosa, eyes, joints, intestine and central nervous system. It has a peculiar geographic distribution, being more prevalent throughout the Mediterranean and the Far East. In general, it affects men more severely and the severity of the disease usually decreases over time (4).

RAS has been identified as the first symptom of Behcet's syndrome (5-8). In 1990, a set of diagnostic criteria was suggested by the International Study Group (ISG) (4). In addition to the mandatory criterion of recurrent oral ulcers (at least three episodes in 12 months), the following are also included: recurrent genital ulcers; ocular lesions such as anterior uveitis or retinal vasculitis; cutaneous manifestations such as erythema nodosum, pseudofolliculitis, papular/pustular lesions or acneiform nodules; and the positive pathergy test (4), which consists of a nonspecific hypersensitivity skin reaction induced by a pinprick performed to look for evidence of this phenomenon (4). Such criteria were revised gave rise to the International Criteria Behcet Disease (ICBD), which included vascular manifestations among the criteria of Behcet’s Disease (BD) (9). Clinically, there are no differences between the oral ulcers presented in patients with RAS and in the syndrome of Behcet, and the clinician should pay attention to the presence of other systemic manifestations that make up the syndrome.

In view of the high prevalence (90-100%) of mouth ulcers in patients with Behcet's syndrome (10), it is extremely important to investigate this condition in RAS, since the latter is a common condition linked to a rare disease which, for many times, may be underdiagnosed. Furthermore, understanding the prevalence of this syndrome in patients with RAS may provide further insight into the pathogenic mechanisms underlying both conditions and contribute to the development of a standardized diagnostic plan, avoiding underdiagnosed cases. Therefore, the purpose of this study was to assess the prevalence of Behcet's syndrome in patients with RAS, through a systematic review of the literature.

## Material and Methods

-Protocol and Registration

This article was reported in accordance with the Preferred Reporting Items for Systematic Reviews and Meta-Analyses (PRISMA) checklist (11) and was submitted to the Prospective Register of Systematic Reviews (CRD42023455163).

-Eligibility criteria and selection process

A specific question guided this systematic review based on the criteria “Condition, concept and population” (CoCoPop): “Does Behcet's syndrome have a significant prevalence in patients with recurrent aphthous stomatitis (RAS)?”

Codition Recurrent aphthous stomatitis

Concept Prevalence studies

Population: Patients with Behçet's syndrome

The following inclusion criteria were used: cross-sectional studies in which a sample of patients with RAS were evaluated and verified the association with Behcet's Syndrome, making it possible to determine the prevalence of the condition. The exclusion criteria included reviews, case reports and studies whose samples consisted primarily of patients with Behcet's Syndrome, with RAS being just one of the clinical manifestations.

-Data Sources and Search Strategies

The search was conducted in May 2024. Two independent investigators (A.B.F.A. and J.S.S.S.S.) selected articles by title and abstract in databases such as Scopus, Medline/PubMed, The Cochrane Library and Web of Science. In addition, a DANS Easy Archive search was performed to access the gray literature and also a manual search in the reference list of the included studies was used as an additional resource to refine the search. In case of disagreement, a third researcher (W.R.S.) was consulted.

The search strategy used in the databases involved the following keywords and MeSH terms: “Aphthous Stomatitis”, “Aphthous Ulcer”, “Aphthous Stomatitides”, “Behcet Syndrome”, “Behcet Disease”, “Adamantiades-Behcet Disease”. All terms were adapted to the different databases and were combined using the Boolean operators “AND” and “OR”. To eliminate duplicates and select included studies, EndNote® software was used or performed manually.

-Data extraction, quality assessment and risk of bias

The articles selected for eligibility were read in full and those that met the inclusion criteria were carefully analyzed and their methodological aspects are described in Table 1. The data extracted from the articles were tabulated and included: author, year and country of study, design of the study, sample size, gender, age, performance of the pathergy test, prevalence of patients with the syndrome and sample collection site.

Methodological quality and risk of bias were assessed using the Joanna Briggs Institute (JBI) Critical Assessment Checklist for Analytical Cross-Section Studies (12) in order to analyze the methodological quality of the included articles. The JBI Critical Assessment Checklist for Analytical Cross-Section Studies features eight questions that consider examination of inclusion criteria, details about participants and study setting, valid and reliable exposure and outcome measures, objective/standard criteria for measuring the condition, identification of confounding factors and appropriate statistical analysis. The questions must have answers according to: “Yes”, “No”, “Uncertain” or “Not Applicable”. “Yes” scores greater than 5, between 3-4, and between 0-2 were classified as high, moderate, and low methodological quality, respectively. Two independent reviewers (A.B.F.A. and J.S.S.S.S) assessed whether each study met the eight items of the checklist, indicating “yes”, “no”, “unclear” or “not applicable”. In case of disagreement, a third evaluator (W.R.S) was consulted to resolve the disagreement.

-Additional Analysis

The inter-rater kappa coefficient was determined to verify the agreement between raters at the time of inclusion of studies from the PubMed/MEDLINE, Web of Science, Scopus and Cochrane library databases.

## Results

- Study selection

**Figure 1:**
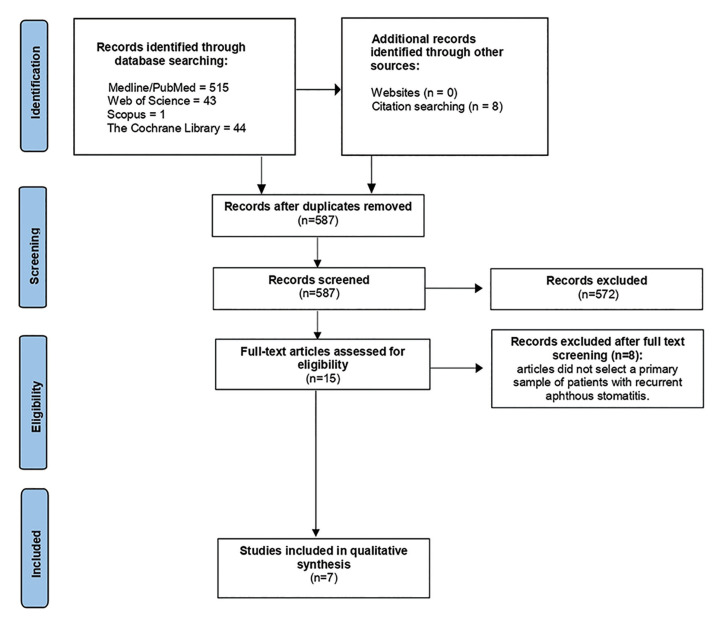
PRISMA based flowchart diagram of literature search.

After searching the databases, 603 titles and research abstracts were found in the database allocated as follows: PubMed (n = 515), Web of Science (n = 43), Scopus (n = 1) and The Cochrane Library (n = 44). No results were found in the DANS Easy Archive, but 8 articles were selected in the manual search. 24 duplicate articles were removed, all titles and abstracts (n = 587) were methodically examined by three researchers and 572 were excluded. A full reading of 15 articles was carried out to verify if they met the inclusion criteria. 7 were included in the systematic review (Fig. 1).

- Description of studies

**Figure 2:**
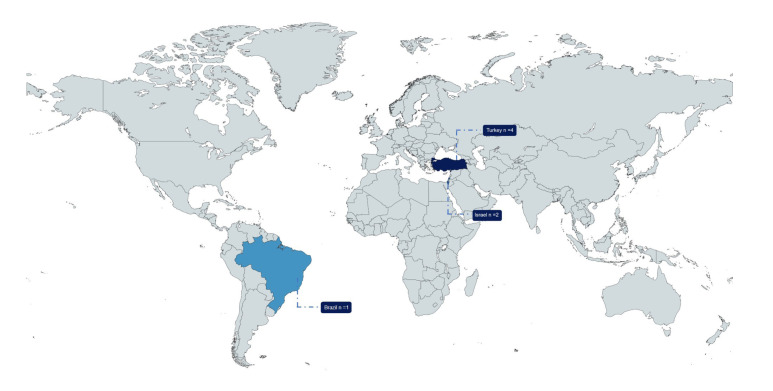
Worldwide distribution of articles that were included in the review.

The characteristics of the included studies are presented in Table 1. All seven were cross-sectional in design, four took place in Turkey, two in Israel and one in Brazil (Fig. 2). The studies were carried out in reference centers, public/community units or epidemiological research in households, allowing broad access to the studied population.

- Assessment of the quality of studies and quality of evidence

Cross-sectional studies were evaluated according to the JBI Critical Assessment Checklist for Analytical Cross-Section Studies (12) (Table 2). Overall, the studies demonstrated high quality, producing positive answers to 5-7 of the 8 questions. Negative responses were associated with the fact that only one of the articles mentioned confounding factors, none described strategies to deal with these factors, and three did not describe the statistical analysis method used.

- Criteria used to diagnose Behcet's Syndrome

All studies, except Bas *et al*. (2016) (13) investigated Behcet's syndrome from the previous screening of patients with recurrent aphthous stomatitis (RAS), through questionnaires, home visits and/or clinical examinations. With the selection of participants with recurrent oral ulcerations, other alterations that could lead to the diagnosis of the referred syndrome were analyzed. Bas *et al*. (2016) (13) randomly selected a representative sample of the population of Tokat (Turkey) aged 20 years and over. Initially, blood tests were carried out and those who had symptoms of BD were subjected to a pathergy test, ophthalmological examinations, in addition to being evaluated for the presence of RAS. From the diagnosis of patients with RAS, it was possible to determine the prevalence of Behcet syndrome in this last condition.

The International Study Group (ISG) (4) was used by all studies as a reference for the diagnosis of Behcet's syndrome. Its set of criteria considers the mandatory presence of recurrent oral ulcers (appearing at least three times a year) and at least two more alterations between genital ulcers, uveitis, cutaneous manifestations and a positive pathergy test.

- Prevalence of Behcet's syndrome in patients with recurrent aphthous stomatitis

**Figure 3:**
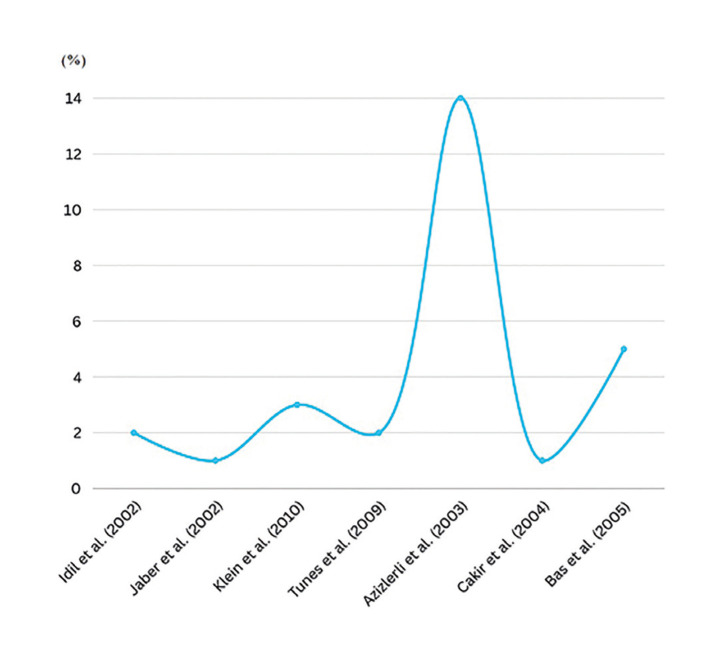
Prevalence of Bechet’s disease in patients with RAS according to the authors.

Idil et. al (2002) (8) presented a prevalence of 1.94%: among 823 individuals with RAS, 16 had the syndrome, with a greater predominance in females; Jaber *et al*. (2002) (14) evaluated 829 participants with RAS and of these, 6 were diagnosed with the syndrome, representing a prevalence of 0.72% and higher frequency in women; Klein *et al*. (2010) (15) showed a prevalence of 3.17% in their study: among 63 individuals evaluated, 2 had the syndrome, with no difference in frequency between the sexes; Tunes *et al*. (2009) (10) showed a prevalence of 2%: among 50 participants with RAS, only one individual was diagnosed with Behcet's syndrome and was male; Azizlerli *et al*. (2003) (16) demonstrated the highest prevalence, corresponding to 14.43%: of the 700 participants, 101 were diagnosed with the syndrome, with a greater predominance of males; Cakir *et al*. (2004) (17) evaluated 124 individuals with RAS, with only 1 diagnosed with the syndrome, without reporting the affected sex, representing a prevalence of 0.81%, and Bas *et al*. (2016) (13) found the second highest prevalence, with 5.5%, and a predilection for females. This data is distributed in Fig. 3.

## Discussion

Based on the analyzed results, there is a wide range of studies evaluating the prevalence of Behcet's syndrome in the Middle East (8,13-17). Of the included studies, only one occurred in the Americas region, in Brazil (12). This may explain, in part, the greater geographic prevalence of this condition in the range that extends from the Mediterranean Basin to the Far East (18) Another considerable fact that may be associated with this higher geographic prevalence corresponds to the large number of marriages between members of the same family: in a study carried out in an Israeli Arab community, 44% of all marriages were consanguineous, which can lead to various genetic diseases and congenital malformations (14).

One of the criteria used for the diagnosis of Behcet's syndrome is the positive pathergy test, which was first described in 1937 by Blobner and reflects an exaggerated cutaneous response to needle trauma due to hypersensitivity, manifesting clinically as an erythematous induration at the site of skin trauma, which may remain as papules or progress to sterile pustules (4). Studies have shown that patients diagnosed with Behcet's syndrome exhibit high positivity in the pathergy test: Idil *et al*. (2002) (12) reported that among 16 participants diagnosed with the syndrome, 13 had a positive pathergy test, corresponding to 81.25%. Azirleli *et al*. (2003) (15) and Cakir *et al*. (2004) (17) found a positive association of 69.31% and 100%, respectively, while the false-negative rate ranged from 2.41% to 12.87% between studies. These data reinforce the reliable results of the pathergy test in the diagnosis of Behcet's syndrome, which may vary according to genetic and environmental alterations, related to the diameter of the needle used, use of corticosteroids and other factors (4).

Variation was observed in the studies regarding the predominance of the syndrome in gender. Idil *et al*. (2002) (8), Bas *et al*. (2016) (13) and Jaber *et al*. (2002) (14) found a greater predominance in females, while Azizlerli *et al*. (2003) (16) and Tunes *et al*. (2009) (10) showed a higher frequency in males, indicating a similar proportion in both genders (19). Regarding age, the studies sought to evaluate individuals over 10/12 years of age, due to the higher prevalence of Behcet's syndrome occurring in young adults, with a mean onset age between 25 and 30 years (17).

Some of the limitations presented concern the lack of clinical follow-up over time in most studies: only Idil *et al*. (2002) (8) reported a follow-up of 6 patients who did not fully meet criteria for Behcet's disease. Another limitation corresponded to the sample studied and the objective of the study: only Tunes et. al (2009) (10) had as its primary objective to investigate the prevalence of Behcet's syndrome in patients with RAS. The other studies aimed only to evaluate the prevalence of Behcet's syndrome in a general population, over 10/12 years of age, in order to include patients with RAS as the first stage of the evaluation, which made it possible to analyze the prevalence in this population. Furthermore, some cases of the syndrome among patients with RAS may have been underdiagnosed, since Behcet's syndrome is clinically diagnosed and some patients may have chronic and asymptomatic uveitis. The studies also did not provide the known differential diagnoses of Behcet's syndrome, such as NeuroBehcet's syndrome, pemphigus and other causes of oral/genital ulcers. Only Bas *et al*. (2016) (13) and Azizlerli *et al*. (2003) (16) reported confounding factors associated with RAS, such as autoimmune diseases, bullous dermatosis, oral malignancies, oral lichen planus, systemic lupus erythematosus, human immunodeficiency stomatitis due to herpes simplex, mucosal ulceration due to dental trauma, among others.

The studies showed a low prevalence, with the lowest corresponding to 0.72% (6:829) in Israel (14), and the highest and most significant 14.43% (101:700) in Turkey (16). Some variables such as sample size and parameters for assessing the RAS condition may have influenced this result: Tunes *et al*. (2009) (10) used a sample containing only 50 participants, which may have reflected in their finding of only one patient diagnosed with the syndrome. Azizlerli *et al*. (2003) (15), for example, did not establish any recurrence limit in the definition of RAS, which may explain a higher prevalence of Behcet's syndrome in their study compared to the others (14.43%); while Jaber *et al*. (2002) (14) obtained the lowest prevalence (0.72%), probably associated with the criteria established for the diagnosis of RAS in their study: at least four recurrences in the last year, with each episode lasting more than seven days.

Recurrent aphthous stomatitis (RAS) remains the most common ulcerative disease in the oral mucosa (19) and constituted the first stage of screening for the diagnosis of Behcet's syndrome in almost all studies (8,10,14-17) as this is a primary and determining criterion for diagnosis, according to the International Study Group (ISG) (4). This fact reinforces the need for studies focused on individuals with recurrent aphthous stomatitis, since, as it is a common condition and there are no clinical differences between oral ulcers presented in systemically healthy and syndromic individuals, it can be underestimated and not be deeply evaluated for the presence of other clinical manifestations that are part of Behcet's syndrome, resulting in an underdiagnosis. Furthermore, genetic, geographic and environmental factors influence its distribution, making more population-based studies necessary, especially in the Americas, where research is scarce, so that its real worldwide prevalence can be established.

## Conclusions

In view of the above, it can be concluded that the prevalence of Behçet's syndrome in patients with recurrent aphthous stomatitis (RAS) is generally low, with a similar frequency in both sexes and more common in the range of countries that extends from the Mediterranean Basin to Far East.

## Figures and Tables

**Table 1:** Summary of the characteristics of included studies.

Author, Year, Country	Study design	N RAS	Prevalence of Behcet's syndrome in RAS	Age (yearsold) BD	Positive skin pathergy test	Gender BD(M/F)	Sample collection site
Idil et. al. (2002) (8) Turkey	Cross-sectional study	823	1,94% (N= 16)	24~67	Total = 10% (N=82) BD=13	5/11	Park Primary Health Care Center
Jaber et.al (2002) (16) Israel	Cross-sectional study	829	0,72% (N= 6)	-	1:2	1/5	Paediatric centre
Klein et al. (2010) (17) Israel	Cross-sectional study	63	3,17% (N=2)	22 and 45	Not done	1/1	Community clinics
Tunes et al. (2009) (12) Brazil	Cross-sectional study	50	2% (N=1)	35	None	1/0	Reference stomatology service
Azizlerli et al. (2003) (18) Turkey	Cross-sectional study	700	14,43% (N=101)	14~68	Total= 83 BD= 70	52/49	Epidemiological research in homes
Cakir et al. (2004) (19) Turkey	Cross-sectional study	124	0,81% (N=1)	-	Total= 4 BD = 1	-	Epidemiological research in rural region
Baş et al. (2016) (15) Turkey	Cross-sectional study	252	5,5% (N=14)	-	57%	M<W	Family Physician Units

N:sample; RAS: recurrent aphthous stomatitis; BD: Behçet disease; M: male; F: female; - : not reported.

**Table 2:** Analysis of the risk of bias of the articles included in the review according to JBI Critical Appraisal Checklist for Analytical Cross-Sectional Studies.

Questions	Idil et al. (2002) (8)	Jaber et al. (2002) (16)	Klein et al. (2010) (17)	Tunes et al. (2009) (12)	Azizlerli et al. (2003) (18)	Cakir et al. (2004) (19)	Baş et al. (2016) (15)
Were the criteria for inclusion in the sample clearly defined?	Y	Y	Y	Y	Y	Y	Y
Were the study subjects and the setting described in detail?	Y	Y	Y	Y	Y	Y	Y
Was the exposure measured in a valid and reliable way?	Y	Y	Y	Y	Y	Y	Y
Were objective, standard criteria used for measurement of the condition?	Y	Y	Y	Y	Y	Y	Y
Were confounding factors identified?	N	N	N	N	Y	N	Y
Were strategies to deal with confounding factors stated?	N	N	N	N	N	N	N
Were the outcomes measured in a valid and reliable way?	Y	Y	Y	Y	Y	Y	Y
Was appropriate statistical analysis used?	Y	U	Y	U	Y	U	Y
Total	6	5	6	5	7	5	7

Abbreviations: N: No; Y: Yes; U: uncertain.

## Data Availability

Data will be available on request.
